# Safety, feasibility, and application of intraperitoneal gas-based hyperthermia beyond 43°C in the treatment of peritoneal metastasis: An *in-vivo* pilot study

**DOI:** 10.3389/fonc.2022.953920

**Published:** 2022-10-11

**Authors:** Agata Diakun, Tanja Khosrawipour, Agata Mikolajczyk-Martinez, Jakub Nicpoń, Simon Thelen, Zdzisław Kiełbowicz, Przemysław Prządka, Bartłomiej Liszka, Joanna Kulas, Kacper Zielinski, Shiri Li, Hien Lau, Wojciech Kielan, Veria Khosrawipour

**Affiliations:** ^1^ 2nd Department of General Surgery and Surgical Oncology, Wroclaw Medical University, Wroclaw, Poland; ^2^ Department of Surgery (A), University-Hospital Düsseldorf, Heinrich-Heine University, Düsseldorf, Germany; ^3^ Medical Faculty, Heinrich-Heine University Düsseldorf, Düsseldorf, Germany; ^4^ Department of Biochemistry and Molecular Biology, Faculty of Veterinary Sciences, Wroclaw University of Environmental and Life Sciences, Wroclaw, Poland; ^5^ Department of Surgery, Faculty of Veterinary Sciences, Wroclaw University of Environmental and Life Sciences, Wroclaw, Poland; ^6^ Department of Orthopedics and Trauma Surgery, Medical Faculty, Heinrich-Heine University, Duesseldorf, Germany; ^7^ Department of Anaesthesiology, Wroclaw Medical University, Wroclaw, Poland; ^8^ Division of Colon and Rectal Surgery, Department of Surgery, New York Presbyterian Hospital- Weill Cornell College of Medicine, New York, NY, United States; ^9^ Department of Surgery, University of California, Irvine, Irvine, CA, United States; ^10^ Department of Surgery, Petrus-Hospital Wuppertal, Wuppertal, Germany

**Keywords:** intraperitoneal, heat, hyperthermia, peritoneal metastases, gas based

## Abstract

**Background:**

43°Celsius (C) is currently the highest temperature used in the treatment of peritoneal metastasis (PM). Despite sufficient data on water- based hyperthermic solutions in PM treatment, there is currently no information on gas-based hyperthermia extending beyond 43°C. This study is the first to provide *in-vivo* data on different organ systems during and after intraperitoneal gas-based hyperthermia beyond 43°C. The aim of this study is to explore *in-vivo* feasibility, safety, and efficacy of this novel concept from a biological perspective.

**Methods:**

For this study, three swine were subjected to laparoscopy and subsequent gas-based intraperitoneal hyperthermia at 48°, 49° and 50°C under a high-flow air stream. Intraoperative data from multiple temperature sensors were analysed. Additionally, intraoperative anaesthesiologic and gasometrical data was analysed. Postoperatively, swine were monitored for one week and laboratory work-up was performed on postoperative days 1, 3 and 7.

**Results:**

During gas-based intraperitoneal hyperthermia, anesthesiologic parameters did not exhibit critical values. No intra- or postoperative complications were observed. Distinct temperature measurements on the skin, cystohepatic triangle and esophagus did not display any temperature increase. Postoperative laboratory workup did not show any changes in hemoglobin, white blood cell count, platelets, or kidney function.

**Discussion:**

Based on our data, there are no safety concerns for the application of gas-based hyperthermia between 48 - 50°C. In fact, no critical systemic temperature increase was observed. With respect to possible limitations, further *in-vivo* studies are required to evaluate whether gas-based intraperitoneal hyperthermia may be a therapeutic option for PM patients.

## Introduction

The management of disseminated peritoneal metastasis (PM) remains a tremendous challenge in surgical oncology. PM is an aggressive disease with a poor overall prognosis, mostly originating from gastrointestinal tract or gynaecological tumour cells. Depending on the extent of PM, median survival is estimated at 3.7 - 9.8 months after diagnosis (1, 2). Systemic treatments such as intravenous chemotherapy have displayed limitations in changing the overall PM outcome. This is attributed to subtherapeutic chemotherapeutic concentrations in the peritoneal tissue due to systemic drug loss (3, 4). Due to these shortcomings, locoregional concepts have been considered as a more promising alternative to overcome current limitations in PM management. The most effective of these concepts, cytoreductive surgery combined with hyperthermic intraperitoneal chemotherapy (HIPEC), has demonstrated efficacy in a selected group of patients (5, 6). Many studies have described the beneficial effects of local hyperthermia in PM management (7–9), and hyperthermia has shown to increase the response rate of cancer cells to chemo- and radiotherapy (10–13). However, liquid-based hyperthermia also exhibits limitations. In the HIPEC setting, medium perfusate and core body temperatures usually remain at around 40°Celsius (C) (14). An increase in central body or total organ temperature is not desirable and should be avoided. In fact, a recent study by Goldenshluger et al. demonstrated that increases in core body temperature served as a positive predictor for postoperative complications following HIPEC procedures (15). Thus, further increasing applied heat to the peritoneal surface is limited by this countervailing factor. A recent study by Diakun et al. demonstrated that intraperitoneal (i.p.) hyperthermia could be a feasible option to increase applied temperatures far beyond 43°C using a gas-based approach (16). In the presented model, the aim was to investigate whether gas-based intraperitoneal hyperthermia could be a feasible and safe option. Currently, further attempts have been made to analyze this approach. By means of an *in-vivo* swine model, we aim to closely investigate if gas-based hyperthermia exceeding 43°C can be safely applied *in-vivo*, and whether there is an indication of systemic or postoperative complications with respect to organ functions.

## Materials and methods

### *In-vivo* swine model

Using a diagnostic laparoscopy setting with a high-flow air stream of 15 liters per minute (l/min), three 65-day-old swine (Polish white flod) were subjected to gas-based i.p. hyperthermia at 48°, 49° and 50°C, respectively. The swine were part of a multicenter and multinational research study on peritoneal hyperthermia and dehydration called “Intraperitoneal gas-based Hyperthermia and Dehydration”. All animals received humane care in compliance with the Guide for the Care and Use of Laboratory Animals as published by the National Institutes of Health.

### Pre-laparoscopic setting

Gas-based i.p. hyperthermia was delivered *via* a laparoscopic approach using a high-airflow system with compressed and filtered room air. No further additions were made to the composition of applied air.

Following compression, air flowed through a bacterial filter system (Cytiva Whatman™ HEPA-Vent Filter, Thermo Fisher Scientific, Walthman, USA) and was redirected through tubing *via* a heated water bath system which heated the passing air to the desired temperature level. At the tube’s exit site, the temperature was again monitored to ensure exact air temperature calibration. The tube’s exit site was placed into a 10 mm trocar. The subsequent surgical procedure was performed under general anaesthesia. All swine were premedicated with an intramuscular injection of midazolam (0.3 mg/kg, WZF Polfa S.A., Poland), medetomidine (0.02 mg/kg, Cepetor 1 mg/ml, CP-Pharma Handelsgesellschaft, Germany) and ketamine (9 mg/kg, Ketamina 100 mg/ml, Biowet Puławy sp. z o.o., Poland) mixture. Analgesia was performed with Propofol at 1mg/kg. Swines were intubated and further anaesthesia was continued with isoflurane 1%. Additional analgesia was provided with fentanyl 2µg/kg and crystalloid fluid at 0,2-0,3 µg/kg/min.

### Surgical setting

**Figure 1:**
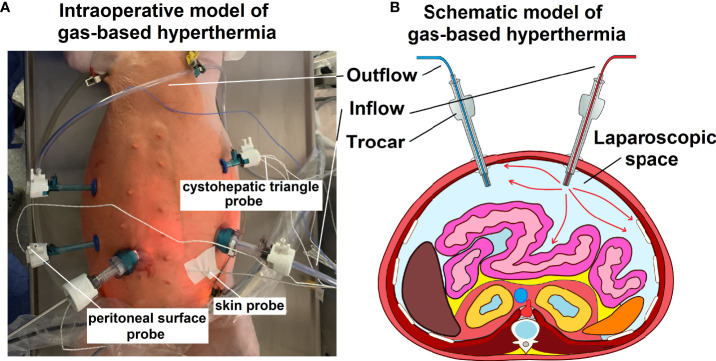
**(A)** Intraoperative model of gas-based i.p. hypertermia in an *in-vivo* swine model. Multiple trocars are placed for visual control, including at the in- and outflow and the entrance point for the temperature sensors. **(B)** Schematic model (transverse section) of the abdominal cavity including the in- and outflow trocar system.

Swines were placed in a supine position. An infra-umbilical mini-laparotomy was performed and another one at about 8 cm distance to the first one. A 10 mm trocar (Kii^®^Balloon Blunt Tip System, Applied Medical, Rancho Santa Margarita, CA, USA) was inserted while multiple 5 mm trocars were placed at the other sites following insufflation (**Figure 1A**). The abdominal cavity was insufflated with filtered room air through a tube entering the central 10 mm trocar (**Figure 1B**). An initial diagnostic check-up was made *via* laparoscopic imaging using a 5 mm camera system (Karl Storz 5mm/30° Laparoscope/Tuttlingen, Germany) *via* a 10 mm trocar. After visual confirmation and placement of multiple temperature sensors, the high-flow air stream was turned on at 15 l/min for a total of 45 minutes. A wide range of temperature and hygrometric sensors (Digital thermometer, FisherbrandTM Tracebale, Pittsburgh, USA) were placed. These allowed for temperature measurements during the procedures. Data from four of these temperature sensors were included in this study. One sensor was directly placed on the peritoneum of the lower left quadrant. One sensor was placed in the cystohepatic triangle, another one was placed and taped onto the skin of the abdomen in the periumbilical region and a final one was placed in the oesophageal area by the anaesthesiologist. During each procedure, a total of three arterial gasometric measurements were performed at the start of the procedure, 20 minutes into the procedure and shortly before extubating.

### Postoperative management

Postoperatively, swines were observed for 7 days before they were euthanized. During the observation period, daily clinical evaluations were conducted with regards to changes in behavior, eating habits, indications of pain or discomfort and evaluation of the trocar wounds. Furthermore, a blood work-up was performed on postoperative days 1, 3 and 7. Red blood count, hemoglobin, white blood cell count and platelets were quantified. Levels of creatinine, blood urea nitrogen, total protein and albumin were also collected. Furthermore, electrolytes such as sodium, potassium, chloride, as well as C-reactive protein (CRP) levels were measured. After euthanization, laparotomy was performed to evaluate possible signs of macroscopical changes, perforation and or postoperative adhesions.

## Results

### Intraoperative parameters and temperature measurements

**Figure 2:**
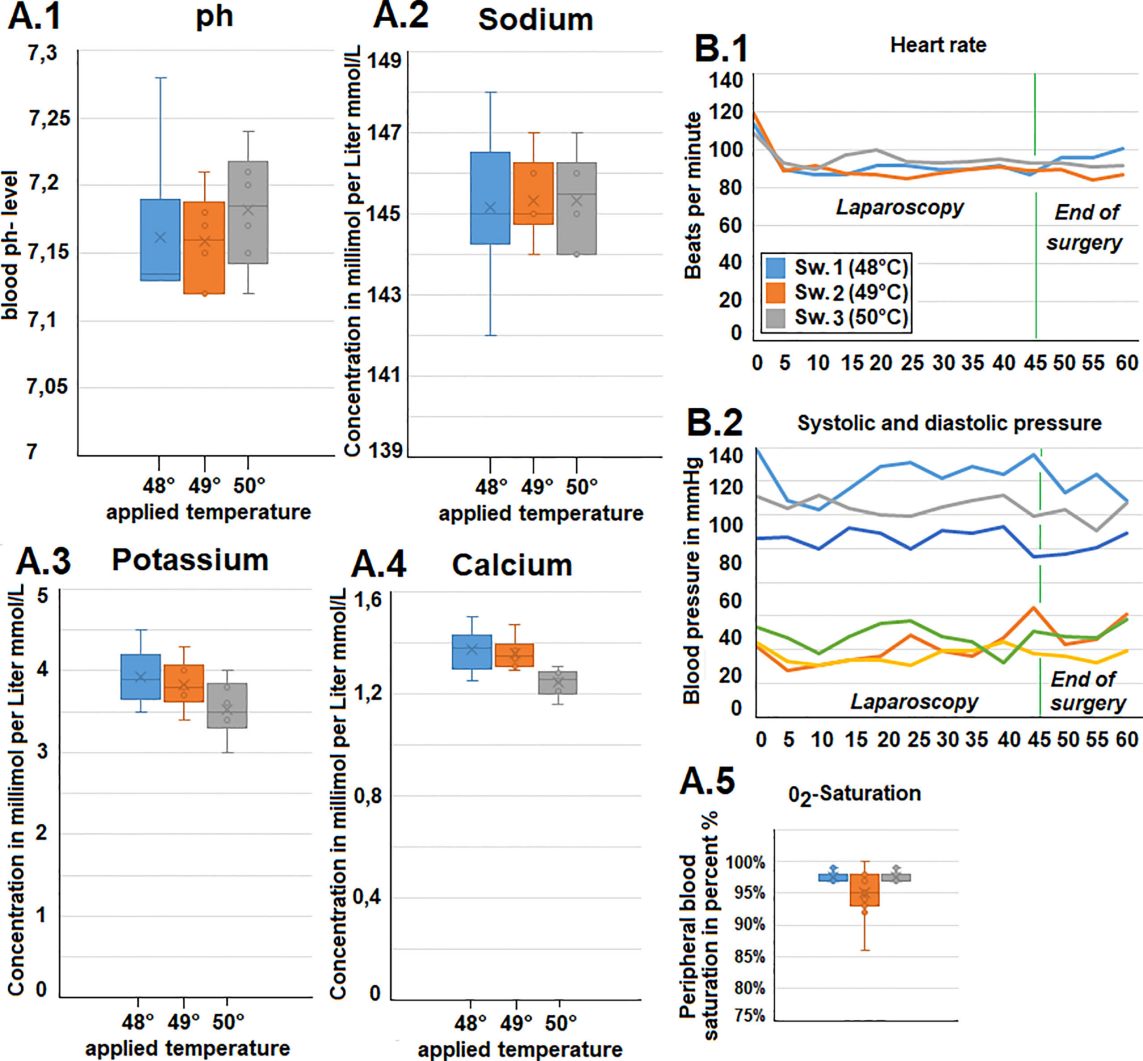
**(A)**. Intraoperative gasometric data. 1) pH-data, 2) plasma sodium level, 3) Plasma potassium level and 4) Plasma calcium level. 5) Intraoperative blood oxygen (0_2_) saturation. **(B.1)** Intraoperative heart rate and **(B.2)** systolic and diastolic blood pressure (via invasive measurement).

**Figure 3:**
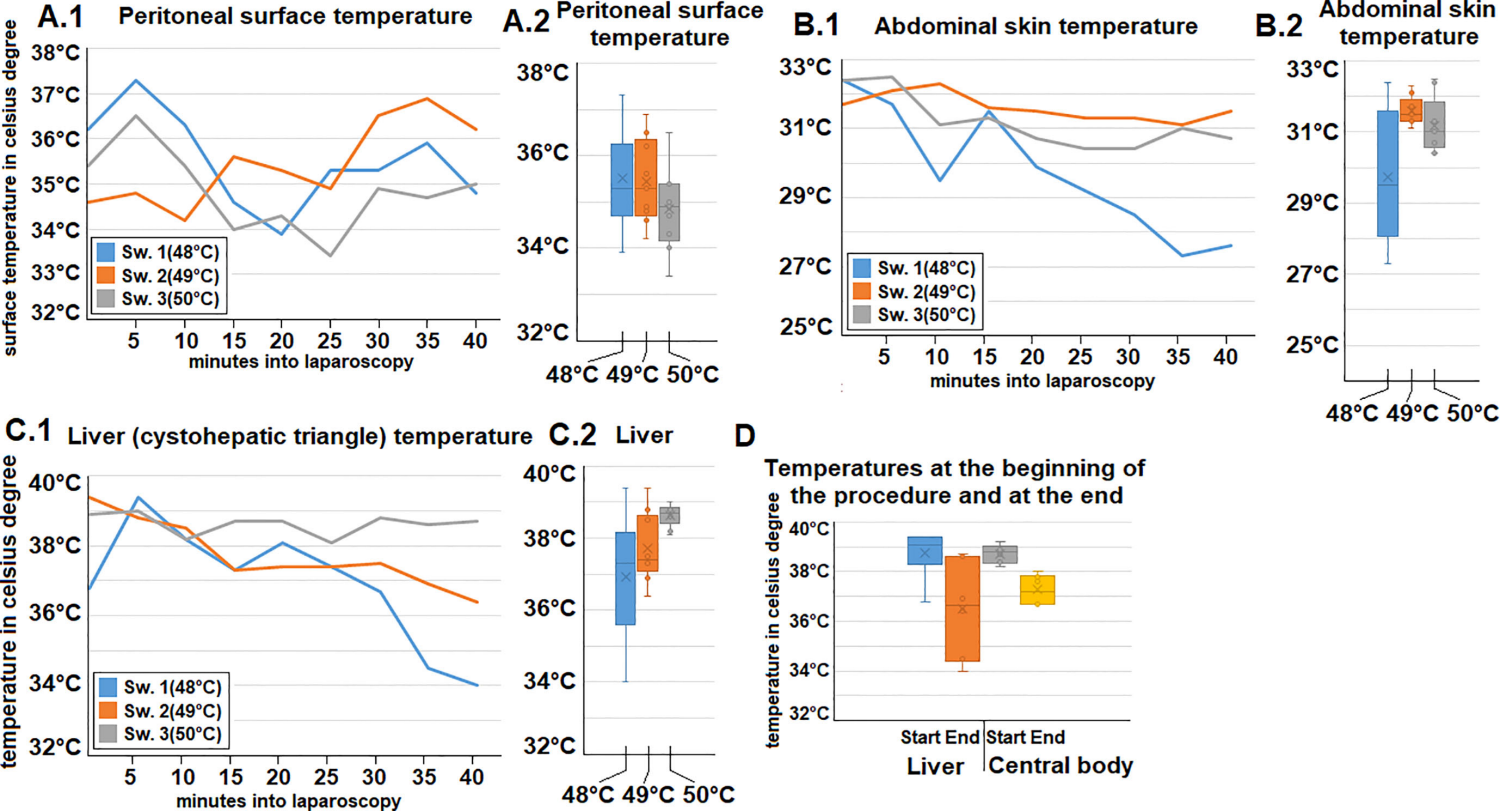
Intraoperative temperature measurements during gas-based i.p. hyperthermia at distinct sites presented for each swine **(A.1, 2)** at the peritoneal surface **(B1, 2)** Abdominal skin, **(C1, 2)** temperature of the “liver” (cystohepatic triangle). **(D)** Measured temperatures at the beginning and end of gas-based i.p. hyperthermia in all swine of the “liver” (cystohepatic triangle) and core body temperature (measured in the oesephageal probe).

Ph., sodium, potassium, and chloride serum levels were measured. These parameters remained mostly constant for each swine within a narrow range (**Figures 2A1–4**). Additionally, both heart rate and blood pressure remained mostly constant despite some fluctuation during the procedure. However, while blood pressure remained constant, an initial drop in heart rate was observed within the first few minutes after initiating the operative procedure (**Figures 2B1, B2**). Oxygen saturation mostly remained constant with only a short intraoperative drop observed in one swine (49°C/**Figure 2A5**). Peritoneal surface temperature was measured during the entire procedure (**Figures 3A1, A2**). While the temperature fluctuated within 5°C, it always remained below 38°C (**Figure 3A2**). Among the swine, the abdominal temperature showed a higher degree of fluctuation (**Figure 3B2**). The skin temperature of one of the swine (48°C) dropped from an initial 33°C to below 28°C during the procedure (**Figure 3B1**). The “liver” temperature measured in the cystohepatic triangle showed a similar behaviour. In two swine, temperatures dropped during the procedure (from 37°C to 34° C° and 39°C to ca. 36°C, respectively, **Figures 3C1, C2**). As for the liver and core body temperature (oesophageal temperature), a slight temperature decrease was noticed from the beginning until the end of the procedure (**Figure 3D**).

### Postoperative parameters and laboratory test analysis

**Figure 4:**
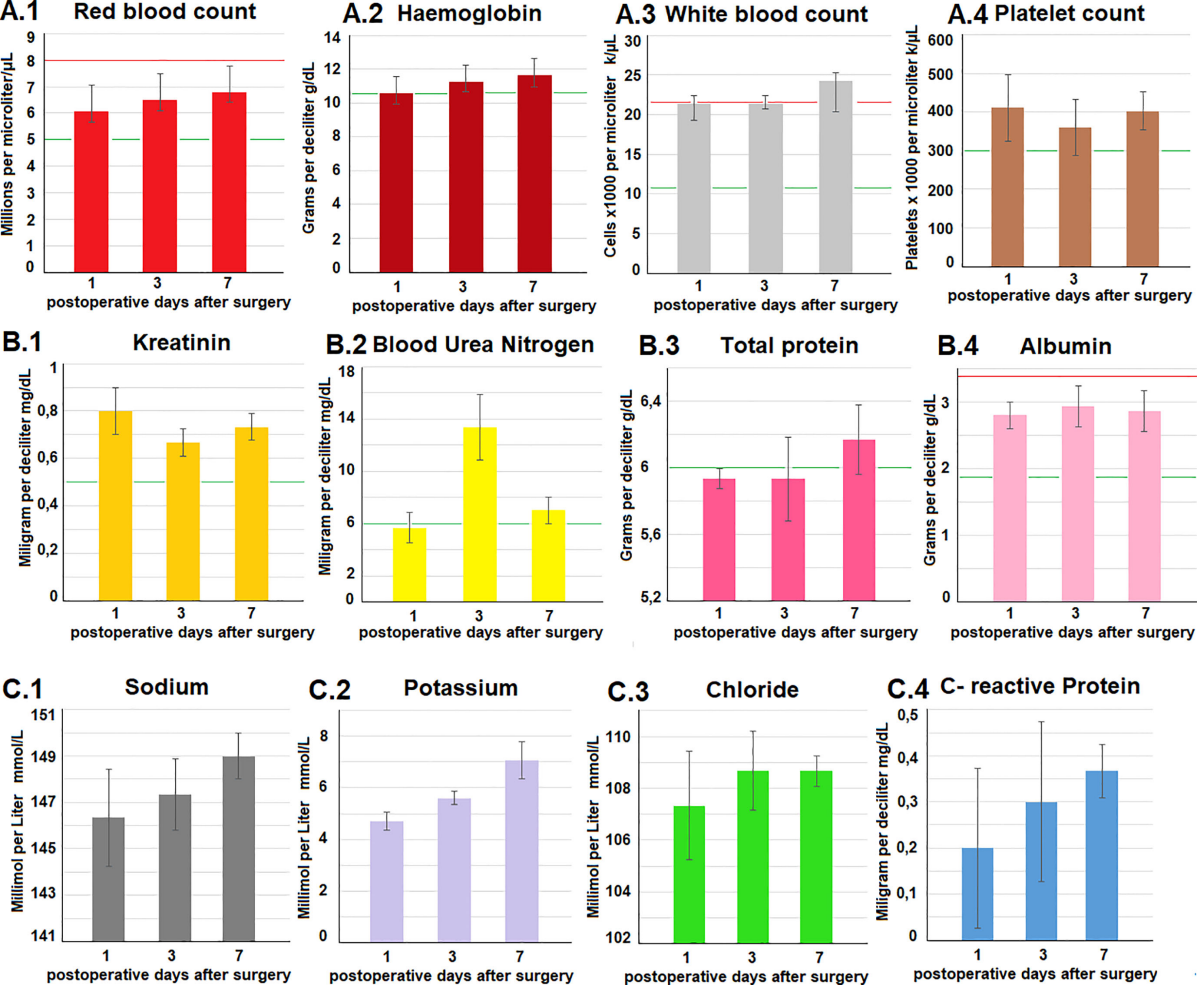
**(A–C)** Blood work-up on postoperative days 1, 3 and 7. Testing included a wide range of parameters. The red (upper) and green lines (lower) in each diagramm demonstrate physiological reference values of the analyzed parameters.

**Figure 5:**
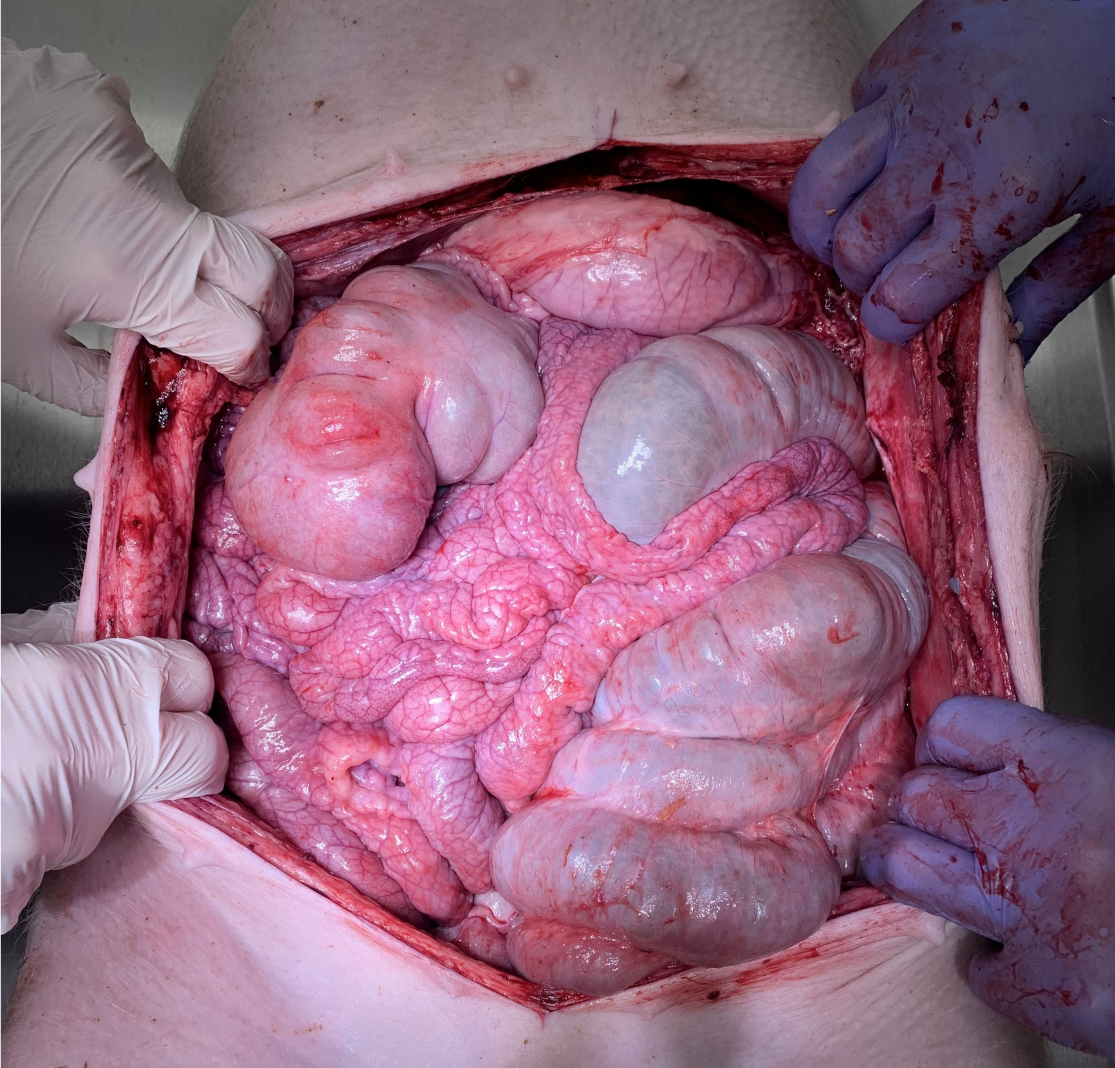
Macroscopic view during postoperative autopsy (day 7). No indication of perforation, adhesions or ascites within the abdominal cavity.

Postoperatively, swine were observed for a total of 7 days before euthanization. No intraoperative complications were detected. Daily clinical postoperative evaluations did not show any changes in behaviour, eating habits, indication of pain or discomfort. All trocar wounds displayed appropriate wound healing. Blood work-up was performed on postoperative day 1, 3 and 7 and did not reveal any concerning pathological signs. Red blood cell count, hemoglobin, platelet count, creatinine, total protein and albumin, as well as sodium chloride and CRP remained within the physiological range and did not show any fluctuation during the study period (**Figure 4**). Potassium levels slightly increased up to day 7, and blood urea nitrogen peaked at day 3 while still remaining within physiological levels. As previously mentioned, CRP always remained below 0.5 mg/dl while a slight elevation in white blood cell count was noted. Again, white blood cell count reached the upper level and was still considered within the physiological range. Autopsy did not show any signs of organ perforation, adhesions, or ascites (**Figure 5**). Small superficial petechia were visible at a few distinct spots.

## Discussion

Cancer cells in the peritoneal cavity usually originate from either gastrointestinal or gynecological cells. Thus, they are used to a stable fluid and temperature environment. This aspect remains unchanged, regardless of mutations in the cancer cell genome (17). Consequently, changing the basic biology of the abdominal cavity might halt tumor progression to a significant degree. Heating and potentially even dehydrating the peritoneal cavity *via* application of continuous gas-based i.p. hyperthermia may serve as a tool to alter basic biology in the human body (16).

The idea of using new physical principles in the treatment of PM and other surface malignancies is promising and has been attempted several times (18–20). Some of these attempts, including irradiation (21–23), high-intensity ultrasound (24, 25) and nanoparticles (26), have been created and extensively tested. Limitations in these approaches are related to the aggressive behavior of PM as well as limited efficacy of i.p chemotherapy even in local applications. However, hyperthermia combined with chemotherapy and CRS has already demonstrated its clinical relevance and impact on the overall outcome in PM management (6–8). How an additional temperature increase beyond 43°C may impact PM management must be further evaluated. Although the inflow temperature exceeds 43°C, we did not detect temperature levels extending beyond 43°C in the organ or at different points within the abdominal cavity. Therefore, the question remains as to whether the occurring thermodynamic energy transfer has an actual effect on the peritoneal surface or if no critical temperature increase is detectable at all. The sensitivity of cancer cells to hyperthermia has already been well demonstrated (27, 28). Moreover, hyperthermia has shown to increase the response rate of cancer cells to chemo- and radiotherapy (10–13). Recently, an enhanced cytotoxic effect of dehydration on colon cancer cells combined with hyperthermia has been described (16). In fact, there are some indications that some of the effects of hyperthermia in the management of local progressive cancer diseases might also be related to the occlusion of neo-vascular tumor structures (29–31)”.

Our data on gas-based hyperthermia beyond 43°C does not indicate any signs of intraoperative or postoperative complications. Blood parameters did not display any indications for possible internal bleeding, platelet depletion, or infections. Additionally, no postoperative complications or organ failure were observed. Moreover, there are no indications for renal issues or protein disbalances in the porcine serum. The blood workup reflects the clinical picture which was void of any complications. Intraoperative measurements did not detect any critical temperature increase. A slight cooling effect during the procedure is attributed to anaesthesiology and supine positioning on a metal-based surgical table.

At this point, it is safe to assume that the application of gas-based i.p. hyperthermia is feasible. While the number of investigated swine does not warrant any conclusive evaluation on potential side effects or complications associated with this concept, this study offers insight into important *in-vivo* aspects of basic feasibility, safety as well as specific characteristics of this novel method. This approach could be combined with the application of chemotherapy, e.g. pressurized intraperitoneal aerosol chemotherapy (PIPAC). However, there are key differences in these applications. During the conventional PIPAC approach, aerosolized chemotherapy is defacto applied in a closed cavity whereas in the presented approach, chemotherapy is applied in a continuous flow system. At the end of gas-based hyperthermia, subsequent PIPAC could be applied. A parallel application is technically challenging since gas turbulence would interfere with chemoaerosol sedimentation. Additionally, management of a high flow, chemoaerosol loaded gas outflow would be demanding on many levels. Further research on this innovative concept is required to evaluate possible *in-vivo* complications and assess its antitumoral effects and benefits for PM management.

### Limitations

Due to the incalculable potential side effects related to the novelty of this pilot study the initial number of swine was kept small to meet ethical and safety concerns. Therefore, the statistical power of this temperature escalation study is limited. The swine model can only partially reflect the conditions and effects on a human. Additionally, the observation period does not fully cover potential complications which might set-in after one week, e.g. adhesions or late perforations. It is important to remember that an additional evaluation of cytoreductive surgery and chemotherapy combined with the presented procedure might also change safety evaluations and could cause concerns that are currently not observed in our model. Hyperthermia’s cellular effects on cancer cells and peritoneal tissue should be further investigated using *in-vitro* and *in-vivo* studies, respectively.

## Data availability statement

The original contributions presented in the study are included in the article/supplementary materials. Further inquiries can be directed to the corresponding author.

## Ethics statement

Experiments were approved (Nr 030/2021/P2) by the Ethics Committee of Wroclaw University of Environmental and Life Sciences, Wroclaw, Poland as well as the local Board on Animal Welfare.

## Author contributions

AD: Study design, laboratory analysis, data acquisition, editing. TK: Study design, conception of the study and manuscript drafting. AM-M and JN: Study design, laboratory analysis, data acquisition. ST, ZK: Manuscript drafting and critical revision for important intellectual content of the manuscript. BL: Study design, laboratory analysis. PP, JK, and KZ: laboratory analysis, data acquisition. SL, HL, and WK: Drafting and critical revision for important intellectual content of the manuscript. VK: Supervision on study design, laboratory analyses, conception of the study and manuscript drafting. All authors contributed to the article and approved the submitted version.

## Funding

This study was funded by institutional funds from the participating Departments. No specific funds or grants were applicable.

## Acknowledgments

We thank Dr. Thomas Siegert from the Max-Planck-Institute of Extra-terrestrial Physics in Garching, Germany for his counselling on aspects of thermodynamics and thermo conduction related to the study.

## Conflict of interest

The authors declare that the research was conducted in the absence of any commercial or financial relationships that could be construed as a potential conflict of interest.

## Publisher’s note

All claims expressed in this article are solely those of the authors and do not necessarily represent those of their affiliated organizations, or those of the publisher, the editors and the reviewers. Any product that may be evaluated in this article, or claim that may be made by its manufacturer, is not guaranteed or endorsed by the publisher.
